# Profiles of Perfectionism and School Anxiety: A Review of the 2 × 2 Model of Dispositional Perfectionism in Child Population

**DOI:** 10.3389/fpsyg.2016.01403

**Published:** 2016-09-14

**Authors:** Cándido J. Inglés, José Manuel García-Fernández, María Vicent, Carolina Gonzálvez, Ricardo Sanmartín

**Affiliations:** ^1^Department of Health Psychology, Miguel Hernandez University of ElcheElche, Spain; ^2^Department of Developmental Psychology and Didactics, University of AlicanteAlicante, Spain

**Keywords:** 2 × 2 model of perfectionism, school anxiety, cluster analysis, Primary Education, socially prescribed perfectionism, self-oriented perfectionism

## Abstract

The 2 × 2 model of dispositional perfectionism has been very well received by researchers of the topic, leading to the creation of new studies that have analyzed the way in which the four proposed subtypes are distinctly associated with measures of adaptation and maladjustment. The goal of this study was to determine the possible existence of four profiles of child perfectionism that are congruent with the subtypes proposed by the 2 × 2 model, and whether these subtypes are associated with school anxiety, in accordance with the hypotheses established by the model. The sample was composed of 2157 students from Spanish Primary Education aged between 8 and 11 years (*M* = 9.60, *SD* = 1.24). The *Child and Adolescent Perfectionism Scale* was used to assess Socially Prescribed Perfectionism and Self-Oriented Perfectionism, and the School Anxiety Inventory for Primary Education was used to measure school anxiety. The results of cluster analysis identified four differential groups of perfectionists similar to the subtypes defined by the 2 × 2 model: Non-Perfectionism, Pure Personal Standards Perfectionism (Pure PSP), Pure Evaluative Concerns Perfectionism (Pure ECP), and Mixed Perfectionism. The four groups presented a differentiable pattern of association with school anxiety, with the exception of Pure PSP and Pure ECP, which showed no significant differences. Participants classified as Non-perfectionists presented the most adaptive outcomes, whereas subjects included in the Mixed Perfectionism group scored significantly higher on school anxiety than the three remaining groups. To conclude, the results partially supported the hypotheses of the 2 × 2 model, questioning the consideration of Self-Oriented Perfectionism as a positive manifestation of perfectionism and showing that it is the combination of high scores in both perfectionist dimensions, Self-Oriented Perfectionism and Socially Prescribed Perfectionism that implies higher levels of school anxiety. These findings should be taken into account when generalizing the 2 × 2 model to child population.

## Introduction

Perfectionism is a complex personality trait of an intra- and interpersonal nature, without a unanimously accepted definition. In fact, the inherent complexity of the construct has led to the existence of multiple conceptualizations of it (Stairs et al., 2012), and there is an intense ongoing debate about the facets that compose it, as well as about its adaptive and/or maladaptive nature (e.g., Flett and Hewitt, 2006; Owens and Slade, 2008). Both perfectionism heads have important implications from an educational point of view, especially due to its differentiating association with motivational variables and achievement-related behaviors and beliefs (Bong et al., 2014).

In an attempt to integrate the conceptualizations of perfectionism, diverse studies (Frost et al., 1993; Dunkley et al., 2000; Stoeber and Otto, 2006) proposed to group the various dimensions attributed to perfectionism into two dimensions, called, predominantly, Personal Standards (PS) and Evaluative Concerns (EC), which have been associated with indicators psychological adaptation and maladjustment, respectively (Stoeber and Otto, 2006). Thus, EC integrates those perfectionist facets associated with fear and concern about making mistakes or being judged negatively by others, a tendency to react negatively to manifestations of imperfection, and with feelings of discrepancy between one’s self-expectations and one’s performance. On another hand, PS includes perfectionist traits such as the tendency to strive and be motivated by achieving perfection and setting excessively high performance standards (Stoeber, 2012). The consideration of these two perfectionist dimensions simplifies the complexity of perfectionism and allows us to compare studies that have used different scales to evaluate it.

Gaudreau and Thompson (2010) developed the 2 × 2 model of dispositional perfectionism, which postulates the existence of four prototypical subtypes of perfectionism, according to the possible combinations of high and low levels of EC and PS: Non-Perfectionism (low EC and low PS), Pure Evaluative Concerns Perfectionism (hereafter Pure ECP; high EC and low PS), Pure Personal Standards Perfectionism (hereafter Pure PSP; low EC and high PS), and Mixed Perfectionism (high EC and high PS). This model is based on the following hypotheses: compared with the Non-Perfectionism subtype, the Pure PSP subtype is associated with healthier results (Hypothesis 1a), with less healthy results (Hypothesis 1b), or alternatively, the results do not differ significantly (Hypothesis 1c); Pure ECP leads to the most maladaptive results of all the subtypes (Hypothesis 2); the Mixed Perfectionism subtype is related to better results than the Pure ECP subtype (Hypothesis 3); and lastly, the Mixed Perfectionism subtype is related to worse results than the Pure PSP subtype (Hypothesis 4). Gaudreau and Thompson (2010) tested their model in a sample of 397 Canadian university students with a mean age 20.39 years, using moderate hierarchical regression methodology. Perfectionism was evaluated using the reduced versions (Cox et al., 2002) of the two Multidimensional Perfectionism Scales (FMPS; Frost et al., 1990; HMPS; Hewitt and Flett, 2004). The results revealed that the Pure PSP subtype was significantly associated with higher academic self-determination, satisfaction, positive affect, and goal progress than the Non-Perfectionist subtype, supporting Hypothesis 1a. In contrast, and in consistency with Hypothesis 1c, no significant differences were found between the two subtypes in negative affect. Likewise, Hypotheses 2, 3, and 4 were also supported by the results of the study. The Mixed subtype was associated with higher levels of negative affect and lower levels of academic self-determination, satisfaction, positive affect, and goal progress than the Pure PSP subtype, and the opposite occurred in the Pure ECP subtype, which was associated with more negative results in terms of maladjustment.

The 2 × 2 model follows a multidimensional conceptua lization of perfectionism and defends that the subtypes cannot be understood without taking into account the continuous distribution of the facets and dimensions of perfectionism. Nevertheless, this model attempted to offer a harmonious perspective between the categorical vs. the dimensional currents of research (Gaudreau, 2013). Thus, various studies have recently tested the hypotheses of the model, most of them with a dimensional approach, using either the moderate hierarchical regression analysis method (Gaudreau and Verner-Filion, 2012; Hill, 2013; Crocker et al., 2014; Damian et al., 2014; Hill and Davis, 2014; Mallison et al., 2014; Méndez-Giménez et al., 2014; Speirs-Neumeister et al., 2015) or structural equations (Franche et al., 2012), but also grouping approaches through cluster analysis (Cumming and Duda, 2012; Li et al., 2014). In addition, the possibility of grouping the perfectionist dimensions into two higher dimensions (EC and PS) has allowed researchers to test the hypotheses of the model using different measures of perfectionism. Thus, for example, we highlight the use of the HMPS (Franche et al., 2012; Gaudreau and Verner-Filion, 2012; Speirs-Neumeister et al., 2015) or its version for children and adolescents: the CAPS (Flett et al., 2000, un published; Damian et al., 2014), the FMPS (Cumming and Duda, 2012) or the simultaneous employment of both (Hill, 2013; Hill and Davis, 2014). In contrast, Li et al. (2014) used the Almost Perfect Scale-Revised (Slaney et al., 2001), whereas Mallison et al. (2014) employed the Sport Multidimensional Perfectionism Scale 2 (Gotwals and Dunn, 2009). Méndez-Giménez et al. (2014) used the *Inventario de Perfeccionismo Infantil* [Child Perfectionism Inventory] (Lozano et al., 2012).

As mentioned, diverse studies tested the hypotheses formulated by Gaudreau and Thompson (2010). Even though some of them use different terms to refer to the subtypes proposed by the model, in the present review, we decided to always use the original terminology proposed by Gaudreau and Thompson (2010) in order to facilitate the comparison of the investigations.

With a sample of 208 Canadian athletes aged between 14 and 28 years, Gaudreau and Verner-Filion (2012) compared the associations between the four perfectionist subtypes and positive affect, subjective vitality, and life satisfaction. The results supported Hypotheses 1c, 2, and 3. However, differences were only observed between the Pure PSP subtype and the Mixed subtype with regard to life satisfaction, partially supporting Hypothesis 4. Likewise, Cumming and Duda (2012), using 194 English dance students aged between 14 and 20 years, found that the participants classified as Pure PSP and the Non-perfectionists did not differ significantly in the levels of social anxiety, negative affect, physical symptoms, and physical and emotional exhaustion, in accordance with Hypothesis 1c but they did differ in positive affect, in favor of the Pure PSP group, thus supporting Hypothesis 1a. Pure PSP obtained significantly lower scores than the Mixed subtype in all the measures of maladjustment but not in positive affect, whose differences did not reach statistical significance, partially supporting Hypothesis 4. Lastly, the results did not support Hypotheses 2 and 3 because the Mixed group and the Pure ECP did not differ significantly in terms of adaptation and maladjustment. In a cross-cultural study, comparing 697 university Canadian students of Asian and European origin aged between 16 and 54 years, Franche et al. (2012) obtained support for Hypotheses 1a, 2, and 3 with regard to academic achievement. However, regarding the degree of school satisfaction, differences were found between the participants of Asian and European origin. Thus, whereas the results for the university students of European origin supported Hypotheses 1a, 2, 3, and 4, there were no significant differences between the Mixed prototype and Pure PSP, or between Pure ECP and Non-Perfectionism in the sample of Asian origin, contradicting Hypotheses 2 and 4.

Subsequently, in a sample of 171 English soccer players aged between 13 and 19, Hill (2013) found support for Hypotheses 1a, 2, 3, and 4 regarding the variables of burnout and a decreased feeling of sport achievement. With regard to physical exhaustion and the devaluation of sport, the results supported Hypotheses 1c and 2. Conversely, they found no differences in the association between the Pure ECP and the Mixed subtype and physical exhaustion, or between the PSP subtype and the devaluation of sport. Recently, Hill and Davis (2014) tested the 2 × 2 model in 238 English coaches aged between 18 and 69 years. The results showed that the Pure PSP subtype was associated with more adaptive results in cognitive reappraisal and control of anger expression, both control-out and control-in, in comparison with the Non-Perfectionism subtype. Conversely, no significant differences were observed in the way in which both subtypes were associated with expressive suppression. Hypotheses 2, 3, and 4 were supported by the comparisons between the perfectionist subtypes and the variables of control-out and -in anger expression; as was Hypothesis 2 in the case of cognitive reappraisal and Hypothesis 4 in the case of expressive suppression. No significant differences were found between the Pure ECP subtype and Non-Perfectionism, or between Non-Perfectionism and Pure PSP, in their relation with cognitive reappraisal, contradicting Hypotheses 2 and 4. Likewise, in the case of expressive suppression, no differences were found between the Pure ECP subtype and Non-Perfectionism. However, there were differences between Pure ECP and the Mixed subtype, but not in the expected direction because the Mixed subtype showed more maladaptive results, contradicting Hypothesis 3.

Subsequently, Crocker et al. (2014) evaluated the hypotheses of the 2 × 2 model by means of a longitudinal design composed of 179 Canadian university athletes aged between 17 and 24 years. They found that the Pure PSP subtype was significantly associated with higher levels than the Non-Perfectionism group in control, challenge and threat appraisal during a competition, as well as in positive affect and goal progress. In contrast, they found no significant differences between these two subtypes for the variables of coping and negative affect. In addition, it was observed that the Pure ECP group only obtained more maladaptive results in the variables of challenge and control appraisal and goal progress. Pure ECP also scored significantly higher than the Mixed group in the control and challenge appraisal, goal progress, and positive affect. Lastly, the Mixed subtype, except for the results for the variables of positive affect and problem- and emotion-focused coping, generally obtained significantly more maladaptive results than Pure PSP. In a study with 345 Chinese employees aged between 19 and 42, Li et al. (2014) found no differences in the total scores of burnout between the Non-perfectionists and the Pure PSP group, or between the Pure ECP and Mixed Perfectionism groups but they did find differences between the Mixed and the Pure PSP groups, thereby supporting only Hypotheses 1c and 4 of the 2 × 2 model. Also, Mallison et al. (2014), using 241 English athletes between 11 and 19 years, found evidence in favor of Hypotheses 1a, 2, 3, and 4 of the 2 × 2 model, about the interaction effects of the variables enjoyment, physical self-esteem, and for most of the dimensions of quality of sports friendship. However, for friendship conflicts, only Hypotheses 1c and 3 were confirmed, and for things in common and conflict resolution, the results only supported Hypotheses 1c, 2, and 3. In Romanian adolescent population aged between 15 and 19 years (*N* = 576), Damian et al. (2014) obtained results supporting Hypotheses 1a, 2, 3, and 4 with regard to positive affect. In contrast, the results for negative affect showed that the Pure PSP and the Non-perfectionist groups did not differ, nor did the Pure ECP group and the Mixed subtype, although differences were observed between the Mixed subtype and the Pure PSP group. Thus, only Hypotheses 1c and 4 were confirmed. In the context of Physical Education, using 331 Spanish adolescents, between 12 and 16 years, Méndez-Giménez et al. (2014) found support for Hypotheses 1a, 2, 3, and 4 regarding the dimensions of physical condition, physical skill, life satisfaction, and positive affect. Nevertheless, support was only found for Hypothesis 1a for group differences in the levels of physical self-concept. Likewise, with regard to general self-concept, the results supported Hypotheses 1c, 2, 3, and 4.

Recently, using 393 high-performing American university students (*M*_age_ = 19.7), Speirs-Neumeister et al. (2015) compared the predictive values of each perfectionist subtype for achievement goals. The results, in general, partially supported the hypotheses of the model. Thus, for example, only Hypotheses 1a and 3 were confirmed for approach goals, whereas for avoidance goals, support was only found for Hypothesis 1b, observing higher levels for the Pure PSP subtype than for Non-Perfectionism. Lastly, Gaudreau (2015) developed a measure called Self-Assessment of Perfectionism Subtypes (SAPS) to assess the four subtypes of perfectionism previously proposed by the 2 × 2 model. The correlational analyses supported Hypotheses 1a, 2, 3, and 4 of the model for the variables of self-determination, academic goal progress, and academic joy, as well as Hypotheses 1c, 2, 3, and 4 for life satisfaction.

The review of the scientific literature of the 2 × 2 model of dispositional perfectionism has revealed diverse limitations. Firstly, regarding the participant’s characteristics, the studies that have evaluated the 2 × 2 model have mainly used samples of university students (Gaudreau and Thompson, 2010; Franche et al., 2012; Crocker et al., 2014; Gaudreau, 2015; Speirs-Neumeister et al., 2015), although they also used athletes and coaches (Gaudreau and Verner-Filion, 2012; Hill, 2013; Hill and Davis, 2014; Mallison et al., 2014), dancers (Cumming and Duda, 2012), actively working adults (Li et al., 2014) and adolescents (Damian et al., 2014; Méndez-Giménez et al., 2014). However, the hypotheses of the model have not been contrasted in child population, an aspect that would be great interest because childhood is considered a key stage for the development of perfectionism (Flett et al., 2002). Also, with the exception of the works conducted by Damian et al. (2014), Li et al. (2014) and Méndez-Giménez et al. (2014) with Chinese, Romanian, and Spanish participants, respectively, the above-mentioned investigations employed American or English population. Nevertheless, diverse studies assert that perfectionism may be influenced by the culture of origin (Marten and Rendón, 2012). As a result, the 2 × 2 model might not be generalizable to other populations with different cultural traits, an aspect about which the work of Franche et al. (2012) cast some doubt.

Thirdly, the viability of the hypotheses of the 2 × 2 model has been proven by comparing the results obtained by the four perfectionist subtypes and diverse variables of adaptation and maladjustment, obtaining, in most cases, results that support the 2 × 2 model, although also some contradictory results. Therefore, further research is required to deepen our knowledge in this sense, analyzing the relation between the perfectionist subtypes and other variables of interest, in order to continue enriching the literature on model.

The present study aims to contribute to solving some of the limitations detected by examining the validity of the 2 × 2 model of Gaudreau and Thompson (2010) in a sample of Spanish students aged between 8 and 11 years. This objective is specified in two parts: (a) to determine whether it is possible to find four child perfectionism profiles that match the subtypes proposed by the 2 × 2 model and (b) to confirm the criterion validity of the identified profiles by contrasting the differences in the mean school anxiety scores reported by each profile. This second goal is of great interest because, although perfectionism has been considered an underlying process that can broadly contribute to the development of anxiety in child population (Affrunti and Woodruff-Borden, 2014), to our knowledge, there are no studies that have previously analyzed the relation between perfectionism and anxiety, specifically school anxiety, understood as a set of symptoms grouped into cognitive, psychophysiological, and motor responses emitted by an individual in school situations that are perceived as threatening and/or dangerous (García-Fernández et al., 2008; Inglés et al., 2015).

Based on prior empirical evidence, it is expected that (a) the results of cluster analysis will reveal the existence four perfectionist profiles characterized by combinations in the scores of Socially Prescribed Perfectionism and Self-Oriented Perfectionism equivalent to the four perfectionist subtypes proposed by the 2 × 2 model, in accordance with the works of Cumming and Duda (2012) and Li et al. (2014), which replicated the subtypes of the 2 × 2 model using cluster analysis. Likewise, regarding the second goal of this study, it is expected that (b) the comparisons of the mean score reported by each cluster in the different factors of school anxiety will support Hypotheses 1a, 2, 3, and 4 of the 2 × 2 model, in accordance with most of the studies that have tested the model of Gaudreau and Thompson (2010).

## Materials and Methods

### Participants

The sample was selected through random cluster sampling, taking as primary units the geographical areas of the province of Alicante: central, north, south, east, and west; and as secondary units, the schools each area (selecting between one and three schools in each area with proportional random sampling). Lastly, the tertiary units were the classrooms. By means of this method, we selected a total 25 schools from urban and rural areas, 19 public schools and 6 private ones, from which four classrooms were randomly chosen, 1 for each course from 3rd to 6th grade of Primary Education, obtaining approximately 95 participants per school. We thereby recruited an initial sample of 2157 students, of whom 83(4.57%) were excluded from the study for not having the minimum reading level required to do the tests, 57 (3.14%) for being repeaters, 97 (5.34%) due to lack of parental consent, and 105 (5.79%) because of errors or omissions in the applied tests. Thus, the final sample was composed of 1815 students aged between 8 and 11 years (*M* = 9.60, *SD* = 1.24), enrolled from 3rd to 6th grade of Primary Education. The ethnic composition of the sample was: 87.65 Spaniards, 6.29% South American, 3.57% Arab, 2.14% European, and 0.35% Asian. The 50.36% of participants were males and the 49.64% females. Regarding the sample distribution across age, it was obtained that the 32.34, 27.99, 18.13% and the 21.54% of participants were 8, 9, 10, and 11 years old, respectively. The Chi-squared test showed the uniform frequency distribution of the eight groups by age and sex (χ^2^ = 6.55, *p* = 0.08).

### Measures

#### Child and Adolescent Perfectionism Scale (CAPS; Flett et al., 2000, un published)

The CAPS was developed from the HMPS (Hewitt and Flett, 2004) and is a 22-item self-report measure of perfectionism in children and adolescents as of age 8. It has two dimensions, Self-Oriented Perfectionism, considered as the application of unrealistic performance standards to oneself and the motivation to be a perfectionist (e.g., “I get upset if there is even one mistake in my work,” “I try to be perfect in everything I do”); and Socially Prescribed Perfectionism, which assesses the belief that your loved ones expect you to be perfect (e.g., “My teachers expect my work to be perfect”). The items are rated on a five-point Likert-type scale, with higher scores indicating more perfectionism.

In its original validation with a population of 247 Canadian students aged between 8 and 17, acceptable scores of internal consistency were found (α = 0.85 for Self-Oriented Perfectionism and α = 0.81 for Socially Prescribed Perfectionism), as well as adequate indexes of temporal stability that ranged between 0.66 and 0.74 for the dimensions of Self-Oriented Perfectionism and Socially Prescribed Perfectionism, respectively. The concurrent and discriminant validity of the scale were also supported by the results of the correlations between the two perfectionist dimensions and diverse measures of adaptation and maladjustment (Flett et al., 2000, un published).

The CAPS is the most used measure of child perfectionism (García-Fernández et al., 2016). Moreover, the scale has been adapted for application in Scottish (O’Connor et al., 2009), Turkish (Uz-Baks and Siyez, 2010), French (Douilliez and Hénot, 2013), Portuguese (Bento et al., 2014), and Chinese (Yang et al., 2015) population. Castro et al. (2004) applied a Spanish translation of the scale in a clinical and non-clinical sample of female adolescents, obtaining adequate rates of internal consistency (α = 0.88 for Self-Oriented Perfectionism, α = 0.87 for Socially Prescribed Perfectionism, and α = 0.89 for the total scale) and temporal stability (*tr* = 0.83). Also, Vicent et al. (2016) obtained appropriate reliability rates for Socially Prescribed Perfectionism dimension (α = 0.88) in a study of 804 Spanish students aged between 8 and 11 years. However, as the scale still has not yet been validated in Spanish population, the Spanish version of the CAPS was established using the back-translation method. Firstly, the original English version of the CAPS was translated into Spanish by a Spanish interpreter with a college degree in English, who was familiar with the English culture. Then, the Spanish translation of the CAPS was back-translated into English by another native Spanish translator with a degree in English language and knowledge of both cultures. The original version was compared with the back-translation and the translators made corrections and drafted the final Spanish translation. No item was deleted or significantly changed during the translation process.

Internal consistency, Cronbach’s alpha, calculated for the present study was 0.84, for the total CAPS, 0.78 for Socially Prescribed Perfectionism, and 0.71 for Self-Oriented Perfectionism.

#### *Inventario de Ansiedad Escolar para Educación Primaria* [Inventory of School Anxiety for Primary Education] (IAEP; García-Fernández et al., 2014)

The IAEP was developed from of the *Inventario de Ansiedad Escolar para alumnos de Educación Secundaria y Bachillerato* [Inventory of School Anxiety for Students of Secondary Education and High School] (García-Fernández et al., 2011) to assess school anxiety in children aged 8–11 years. It consists of 22 items about four school situations that can frequently cause anxiety, and 15 items that present diverse responses of school anxiety. The four situational factors are: (I) School Punishment Anxiety, measuring anxiety in situations of explicit punishment at school or situations that could lead to punishment (e.g., “The teacher asks for my homework and I did not do it”); (II) Victimization Anxiety, which assesses anxiety caused by situations in which the student feels physically or psychologically abused by peers (e.g., “They insult me or threaten me at school”); (III) Social Evaluation Anxiety, which reflects anxiety in anticipation of being negatively judged by others at school (e.g., “Going to the black board”); (IV) School Evaluation Anxiety, in reference to anxiety associated with exams (e.g., “When I’m taking an exam”). The three response scales present cognitive (e.g., “I feel guilty”), behavioral (e.g., “I cannot sit still”), and psychophysiological (e.g., “I breathe faster”) anxiety responses.

García-Fernández et al. (2014) analyzed the validity of the scale by means of exploratory factor analysis (EFA) and confirmatory factor analysis (CFA) in a sample of 1003 Spanish children between 8 and 11 years, supporting the factor structure of the instrument. The levels of reliability were acceptable both for the total scale (α = 0.92) and for the four situational factors (α = between 0.85 and 0.90) and the three response scales (α = between 0.80 and 0.84).

Internal consistency, Cronbach’s alpha, calculated for the present study was 0.92 for the total IAEP, 0.89 for Factors I (School Punishment Anxiety) and II (Victimization Anxiety), 0.87 for Factor III (Social Evaluation Anxiety), 0.88 for Factor IV (School Evaluation Anxiety), 0.74 for the Cognitive Scale, 0.71 for the Behavioral Scale, and 0.73 for the Psychophysiological Scale.

#### Procedure

A meeting was held with the headmasters of the selected schools to explain the purpose of our work and request their collaboration. All the headmasters of the selected schools agreed to participate in our work. After selecting the classrooms, we requested the parents’ written informed consent. Subsequently, we administered the tests to the students during normal school hours, for approximately 40 min, in group format and under the supervision of at least one trained researcher. At the beginning of the administration session of the two instruments, the researcher explained the goal of the work to the participants, focusing on its voluntary and anonymous nature. The participants were treated at all times according to the ethical criteria that govern scientific research.

#### Statistical Analysis

We applied cluster analysis, using the non-hierarchical method of *quick cluster analysis*, which allows previously specifying the number of clusters to be formed, so that only one cluster solution is given, and it also permits moving subjects from one group to another during the grouping process in order to optimize the cluster solution (Clatworthy et al., 2005). In addition, this procedure is considered the most adequate to establish profiles if the sample of participants is sufficiently large (Hair et al., 1998).

The profiles of child perfectionism were defined based on the different combinations of the dimensions Socially Prescribed Perfectionism and Self-Oriented Perfectionism, which were taken as indicators of the two dimensions proposed by the 2 × 2 model (EC and PS, respectively) as in previous studies (Franche et al., 2012; Gaudreau and Verner-Filion, 2012; Damian et al., 2014; Speirs-Neumeister et al., 2015). Before cluster analysis, we standardized the raw scores because the two subscales did not contain the same number of items. In order to replicate the 2 × 2 model, we defined an initial solution of four clusters. According to the criterion of Nordin-Bates et al. (2011) and Cumming and Duda (2012), *z* scores below -0.5 are considered to be low levels; *z* scores between -0.5 and +0.5 moderate, and *z* scores over +0.5 are considered high.

We then carried out various analyses of variance (*ANOVA*) to examine the differences between the four groups identified in the school anxiety dimensions, and thus verify the validity of the hypotheses posed by the 2 × 2 model. Subsequently, in those cases that were statistically significant, *post hoc* tests were performed (*Scheffé* method) to determine between which groups such differences were found. In addition, the effect size or standardized mean difference was calculated (*d* index) to obtain the magnitude of the observed differences, considering values between 0.20 and 0.49 indicators of a small effect size, between 0.50 and 0.79, medium or moderate effect, and values equal to or greater than 0.80 as indicators of a large effect size (Cohen, 1988). All the data analyses were carried out with the SPSS/IBM 22.0 statistical package.

## Results

### Identification of Child Perfectionism Profiles

**FIGURE 1:**
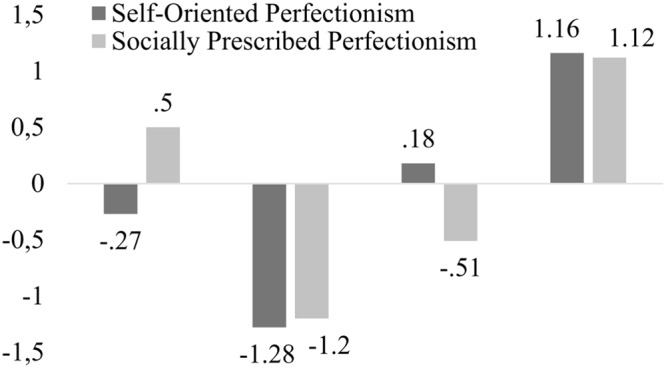
**Graphic representation of the profiles of child perfectionism through cluster analysis**.

According to the above criteria, the first group, which included 470 subjects (25.90%), was characterized by high levels of Socially Prescribed Perfectionism and moderate levels of Self-Oriented Perfectionism. Consequently, this group was called Pure ECP. The second cluster was made up of 381 participants (20.99%) who scored low in the two assessed perfectionist dimensions. Therefore, we decided to call this group Non-Perfectionism. The third cluster included 516 students (28.43%) with moderate scores in Self-Oriented Perfectionism and low scores in Socially Prescribed Perfectionism. This group was called Pure PSP. Lastly, we found a fourth group containing 448 participants (24.68%) with high scores in both scales, which was labeled Mixed Perfectionism (see **Figure 1**).

### Inter-group Differences in School Anxiety

The results of the *ANOVA* revealed the existence of statistically significant differences among the four groups of perfectionists in the mean scores for total school anxiety and in all the subscales. In all cases, the Non-Perfectionism group obtained the lowest means in school anxiety, whereas the Mixed Perfectionism group obtained the highest scores (see **Table 1**).

**Table 1:** Means and standard deviations obtained by the four groups, effect size for each dimension of school anxiety.

	Group 1 Pure ECP		Group 2 Non-Perfectionist		Group 3 Pure PSP		Group 4 Mixed Perfectionismy		Statistical Significance		
Dimensions	*M*	*SD*	*M*	*SD*	*M*	*SD*	*M*	*SD*	*F_(3,1811)_*	*p*	η*^2^*
FI	25.43	14.11	19.47	14.19	25.63	14.97	29.77	16.33	29.240	<0.001	0.152
FII	15.13	14.29	11.75	12.26	16.20	14.36	17.64	16.46	11.057	<0.001	0.120
FIII	8.50	7.92	6.45	7.37	8.15	8.57	10.46	11.35	12.444	<0.001	0.123
FIV	14.06	8.91	11.28	7.93	12.56	8.68	15.26	9.63	14.366	<0.001	0.126
Cognitive Scale	29.48	13.29	23.43	14.16	29.01	14.34	32.45	16.04	24.506	<0.001	0.144
Behavioral Scale	14.85	10.60	11.01	9.69	15.17	10.80	17.88	12.43	24.237	<0.001	0.144
Psycho-physiogical Scale	19.74	14.01	15.41	12.70	19.66	15.12	23.76	16.77	19.334	<0.001	0.135
Total IAEP	63.14	33.46	48.96	32.38	62.55	36.39	73.14	41.15	27.544	<0.001	0.149

*FI, School Punishment Anxiety; FII, Victimization Anxiety; FIII, Social Evaluation Anxiety; FIV, School Evaluation Anxiety.*

The *post hoc* tests (see **Table 2**) revealed statistically significant differences between the Pure ECP group and the Non-Perfectionist group in all the dimensions of school anxiety and in the total IAEP scores, with small effect sizes that ranged between *d* = 0.25 (for Victimization Anxiety) and *d* = 0.43 (for the total scale). The Pure ECP and Pure PSP groups did not differ significantly in school anxiety. In contrast, differences were observed between the Pure ECP cluster and Mixed Perfectionism, with small effect sizes (between *d* = 0.20 for Social Evaluation Anxiety and the Cognitive Scale, and *d* = 0.28, for School Punishment Anxiety). Likewise, the Non-Perfectionism and Pure PSP groups differed significantly, also with small effect sizes that varied between *d* = 0.30, for the Psychophysiological scale, and *d* = 0.42, for the School Punishment Anxiety scale. However, no significant differences were found between the two groups for Social Evaluation Anxiety and School Evaluation Anxiety. No differences were found between the Pure PSP and Mixed Perfectionism groups in the variable Victimization Anxiety, although differences were observed for the rest of factors and scales of school anxiety, with small effect sizes in all cases, ranging between *d* = 0.23 (Social Evaluation Anxiety, Cognitive and Behavioral Scales) and *d* = 0.30 (School Evaluation Anxiety). Lastly, we found the greatest differences between the Pure PSP cluster and Mixed Perfectionism (between *d* = 0.40, for Victimization Anxiety and *d* = 0.67 for School Punishment Anxiety), with moderate effect sizes for the variables of school anxiety.

**Table 2:** *d* Cohen index to *post hoc* contrasts between the mean scores obtained and the four clusters in the factors of school anxiety.

	Pure ECP vs. Non-Perfectionist	Pure ECP vs. Pure PSP	Pure PSP vs. Mixed Perfectionism	Non-Perfectionist vs. Pure PSP	Non-Perfectionist vs. Mixed Perfectionism	Pure PSP vs. Mixed Perfectionism
FI	0.42	n.s	0.28	0.42	0.67	0.27
FII	0.25	n.s	n.s	0.33	0.40	n.s
FIII	0.27	n.s	0.20	n.s	0.41	0.23
FIV	0.33	n.s	n.s	n.s	0.45	0.30
Cognitive Scale	0.44	n.s	0.20	0.39	0.59	0.23
Behavioral Scale	0.38	n.s	0.26	0.40	0.61	0.23
Psycho-physiogical Scale	0.32	n.s	0.26	0.30	0.56	0.26
Total IAEP	0.43	n.s	0.27	0.39	0.65	0.27

*FI, School Punishment Anxiety; FII, Victimization Anxiety; FIII, Social Evaluation Anxiety; FIV, School Evaluation Anxiety.*

## Discussion

The first goal of the present work was to analyze the existence of four profiles of child perfectionism in Spanish population and determine whether these profiles coincided with the perfectionist subtypes identified by Gaudreau and Thompson (2010). Accordingly, the results of the cluster analyses revealed four groups that were characterized by combinations of the scores in Socially Prescribed Perfectionism and Self-Oriented Perfectionism similar to the description of the four perfectionist subtypes proposed by the 2 × 2 model, and coherent with other previous studies of clusters that defined these same perfectionism profiles (Cumming and Duda, 2012; Li et al., 2014).

On another hand, the second goal of this investigation involved comparing the mean scores obtained by the four profiles identified in school anxiety, in order to support the hypotheses previously formulated by the 2 × 2 model. The results revealed statistically significant differences among the four groups, except for between the Pure ECP and Pure PSP clusters, comparing the mean scores obtained on the seven subscales and the total school anxiety score. However, these differences were not as expected. In fact, we found that the results did not support most of the hypotheses of the 2 × 2 model, as we had hypothesized. Specifically, we observed that the Pure PSP group obtained significantly higher scores in school anxiety than the Non-Perfectionism group, confirming Hypothesis 1b, except for the results for Factors III (Social Evaluation Anxiety) and IV (School Evaluation Anxiety), whose differences did not reach statistical significance (supporting Hypothesis 1c), and contradicting previous literature on the model, which had mainly found support for Hypothesis 1a (Gaudreau and Thompson, 2010; Franche et al., 2012; Crocker et al., 2014; Hill and Davis, 2014; Mallison et al., 2014; Méndez-Giménez et al., 2014; Gaudreau, 2015). These findings imply that Non-perfectionist students generally have lower levels of anxiety than students with high levels of PS and low levels of EC. In support of Hypothesis 4, we found that the Pure PSP group differed significantly from the Mixed group, with higher school anxiety scores in the latter group. These findings are in the line of prior works that supported the idea that the Pure PSP group is more adaptive than the Mixed subtype (Gaudreau and Thompson, 2010; Cumming and Duda, 2012; Hill, 2013; Crocker et al., 2014; Damian et al., 2014; Hill and Davis, 2014; Li et al., 2014; Mallison et al., 2014; Méndez-Giménez et al., 2014; Gaudreau, 2015). However, in contrast to Hypotheses 2 and 3, the Mixed Perfectionism group scored significantly higher in school anxiety. That is, contrary to the 2 × 2 model, which postulates that Pure ECP is the most harmful subtype (Gaudreau and Thompson, 2010; Franche et al., 2012; Gaudreau and Verner-Filion, 2012; Hill, 2013; Hill and Davis, 2014; Mallison et al., 2014; Méndez-Giménez et al., 2014; Gaudreau, 2015), the results of this study showed that the Mixed subtype is the most negative, in terms of maladjustment, for the specific case of school anxiety.

In general, the results found seem to indicate that Pure ECP and Pure PSP do not present a differentiable pattern of association with school anxiety, and both are more harmful than the Non-Perfectionism group, which proved to be the most adaptive subtype of the four, whereas the combination of high levels in both dimensions (Self-Oriented Perfectionism and Socially Prescribed Perfectionism) is the most harmful manifestation of perfectionism. These results question the idea that Self-Oriented Perfectionism is a positive personality trait, at least regarding its relation with school anxiety. Certainly, taking into account that Self-Oriented Perfectionism is characterized by a tendency to set unrealistic and even impossible goals and a high motivation to pursue them, as well as the tendency to criticize oneself, it is not surprising that students who obtain moderate and high scores in this dimension (i.e., Pure PSP and Mixed Perfectionism clusters) experience higher levels of anxiety related to the academic setting. In fact, previous studies have also shown in child and youth population that this dimension is positively associated with diverse psychopathological variables such as anxiety (Hewitt et al., 2002; Essau et al., 2012; Nobel et al., 2012), depression (Hewitt et al., 2002; Castro et al., 2004; Christopher and Shewmaker, 2010; Nobel et al., 2012), eating disorders (Castro et al., 2004, 2007; Kirsh et al., 2007; Pamies and Quiles, 2014), a tendency toward shame and guilt (Choy and Drinnan, 2007), narcissisism (Freudenstein et al., 2012), and somatic symptoms (Jungyoon, 2012). Nevertheless, Self-Oriented Perfectionism has sometimes been shown to be associated with adaptive results. Thus, it has also been shown in students of diverse ages that Self-Oriented Perfectionism positively predicts academic achievement (Stoeber et al., 2015) and is positively related to academic self-efficacy and other motivational variables (Bong et al., 2014), to greater optimism about the probability of being successful (Eddington, 2014), and to adaptive problem-solving behaviors (Dry et al., 2015), among others aspects. This dual facet of Self-Oriented Perfectionism has meant that its construct validity has been questioned by diverse studies, which found that the factor structure of the CAPS has a better fit when Self-Oriented Perfectionism is divided into two differentiated scales, Self-Oriented Perfectionism-Efforts and Self-Oriented Perfectionism-Criticism, reflecting, respectively, the positive and negative side of perfectionist introspection (McCreary et al., 2004; O’Connor et al., 2009; Nobel et al., 2012). Therefore, if possible, we recommend replicating the perfectionist subtypes considering Self-Oriented Perfectionism-Efforts as a reflection of the PS dimension, and Self-Oriented Perfectionism-Criticism and Socially Prescribed Perfectionism as a reflection of the EC dimension, as well as analyzing the differences between the groups found, based on various measures of adaptation and maladjustment.

### Limitations and Future Research

Some limitations identified and future lines of research should be mentioned before concluding this study. Firstly, given that perfectionism is multidimensional and lacks a unique definition, it should be taken into account that the use of one or another scale may involve subtle differences in the results. In our case, we chose the CAPS as being the most employed instrument to assess perfectionism in children and adolescents and because it evaluates the dimensions of Self-Oriented Perfectionism and Socially Prescribed Perfectionism, which have proven to be valid and reliable indicators of PS and EC (e.g., Frost et al., 1993; Dunkley et al., 2000; Bieling et al., 2004). Nevertheless, it would be interesting for future works to use other instruments validated in child population, for example, the Adaptative/Maladaptative Perfectionism Scale (Rice and Preusser, 2002; Rice et al., 2004), as the results may differ.

Secondly, only two previous studies have examined the 2 × 2 model through cluster analysis. However, these studies were carried out in very different populations from those employed in the present study. Thus, Cumming and Duda (2012) used English dance students aged between 14 and 20 years, whereas Li et al. (2014) focused on Chinese adults working in departments related to computer science. Therefore, as it has been suggested that perfectionism can vary in intensity and manifestation depending on the domain assessed (e.g., work, studies, physical appearance, sport…) (Stoeber and Stoeber, 2009), the results obtained by these works and by this study should be compared with caution.

Thirdly, regarding the methodological aspect, in this study, we chose by non-hierarchical method for the cluster analysis, as our goal was to replicate the four groups identified by the 2 × 2 model. However, it should be noted that small discrepancies were found with regard to the Pure PSP and Pure ECP groups. That is, whereas according to the 2 × 2 model, the Pure ECP group was characterized by high scores in EC and low scores in PS, and the opposite held true for Pure PSP, in our study, the Pure ECP group was characterized by high scores in EC and moderate scores in PS, and the Pure PSP group, by low scores in EC and moderate scores in PS. This could explain why the differences between these two subtypes in school anxiety did not reach statistical significance. Accordingly, we recommend future studies to determine whether another solution of profiles would better represent child perfectionism.

Fourthly, it is important to mention that, despite our sample size and sampling process guarantee the representability of Spanish students between 8 and 11 years old, it is important that future research consider as well relevant information like socioeconomically origin and previous academic performance, because they could play an important role in the relationship between perfectionism and academic anxiety.

Lastly, this study suggests that approximately one fourth of the child population has high scores in perfectionism and is at risk of presenting high levels of school anxiety. However, the cross-sectional design used limits the interpretation of the results about how perfectionism is associated with school anxiety. Therefore, it would be of interest for future works to solve this limitation by means of a longitudinal design that would allow establishing causal relations between the analyzed variables, as well as determining the negative consequences over time of belonging to one or another perfectionist subtype.

Despite the limitations, this is the first study that analyzes the 2 × 2 model in children from Primary Education (between 8 and 11 years), identifying four distinguishable child perfectionism profiles and comparing their association with school anxiety. The present investigation shows that students characterized by being perfectionists, either of an interpersonal or intrapersonal nature or presenting both forms concurrently, make up more than 70% of the students between 8 and 11 years. Therefore, a considerable number of perfectionist children present greater vulnerability for the development of psychological problems such as, for example, school anxiety. Educational institutions should pay more attention to this problem, intervening more actively. Thus, in the context of a society often characterized by a culture of the hyper-competitiveness (Fletcher et al., 2014), schools must take on the challenge of providing the students with the resilience required to be able to self-regulate, cope with adversity and failures, and to consider their errors as possibilities to improve and not as defects inherent to the person (Flett and Hewitt, 2014; Greenspon, 2014). In short, to form people who are capable of not allowing them to submit to the “tyranny of musts,” that need of chimerical perfection that contributes to the development and maintenance a wide variety of psychiatric disorders (Egan et al., 2011).

## Author Contributions

CI has designed this research. He has also received the study in all its phases. JG-F has designed this research. He has also received the study in all its phases. MV has participated conducting a literature search and writing this manuscript. CG participated conducting a literature search and writing this manuscript. RS has participated performing statistical analyzes.

## Conflict of Interest Statement

The authors declare that the research was conducted in the absence of any commercial or financial relationships that could be construed as a potential conflict of interest.

## Abbreviations

CAPS: Child and Adolescent Perfectionism Scale
EC: Evaluative Concerns
IAEP: Inventario de Ansiedad Escolar para Educación Primaria [School Anxiety Inventory for Primary Education]
FMPS: Multidimensional Perfectionism Scale of Frost et al. (1990)
HMPS: Multidimensional Perfectionism Scale of Hewitt and Flett (2004)
PS: Personal Standards
Pure ECP: Pure Evaluative Concerns Perfectionism
Pure PSP: Pure Personal Standards Perfectionism
